# Genome-wide identification and mining elite allele variation of the Monoacylglycerol lipase (MAGL) gene family in upland cotton (*Gossypium hirsutum* L.)

**DOI:** 10.1186/s12870-024-05297-w

**Published:** 2024-06-21

**Authors:** Zhibin Zhou, Yao Chen, Mengyuan Yan, Shuqi Zhao, Feifei Li, Shuxun Yu, Zhen Feng, Libei Li

**Affiliations:** 1 The Key Laboratory for Quality Improvement of Agricultural Products of Zhejiang Province, College of Advanced Agricultural Sciences, Zhejiang A&F University, Lin’an 311300, Hangzhou, China; 2 Cotton and Wheat Research Institute, Huanggang Academy of Agricultural Sciences, Huanggang 438000, Hubei, China

**Keywords:** Upland cotton, MAGL, Bioinformatics analysis, GWAS, Seed oil content

## Abstract

**Background:**

*Monoacylglycerol lipase* (*MAGL*) genes belong to the alpha/beta hydrolase superfamily, catalyze the terminal step of triglyceride (TAG) hydrolysis, converting monoacylglycerol (MAG) into free fatty acids and glycerol.

**Results:**

In this study, 30 *MAGL* genes in upland cotton have been identified, which have been classified into eight subgroups. The duplication of *GhMAGL* genes in upland cotton was predominantly influenced by segmental duplication events, as revealed through synteny analysis. Furthermore, all *GhMAGL* genes were found to contain light-responsive elements. Through comprehensive association and haplotype analyses using resequencing data from 355 cotton accessions, *GhMAGL3* and *GhMAGL6* were detected as key genes related to lipid hydrolysis processes, suggesting a negative regulatory effect.

**Conclusions:**

In summary, MAGL has never been studied in upland cotton previously. This study provides the genetic mechanism foundation for the discover of new genes involved in lipid metabolism to improve cottonseed oil content, which will provide a strategic avenue for marker-assisted breeding aimed at incorporating desirable traits into cultivated cotton varieties.

**Supplementary Information:**

The online version contains supplementary material available at 10.1186/s12870-024-05297-w.

## Introduction

Cotton stands as an indispensable fiber crop, with ongoing research primarily targeting the enhancement of its fiber quality and yield. Recognized as a crucial oilseed crop [1], cottonseed exhibits a notable abundance of unsaturated fatty acids and essential fatty acids, with linoleic acid content ranging from 28.24% to 44.05% [2]. Wherein, particularly the polyunsaturated fatty acids, plays a crucial role in mitigating disease risks. With its negative carbon profile, leveraging cottonseed oil as a sustainable energy source promises a substantial cut in CO_2_ emissions compared to conventional fossil fuels, marking a stride towards environmental sustainability [3]. Furthermore, the by-products of cotton hold immense potential in various applications, serving as vital components for feed protein and the cultivation medium for edible mushrooms, thus presenting expansive prospects. This versatility not only mitigates the imbalance between grain and cotton production triggered by population surge and dwindling farmland in China but also underscores the imperative of advancing research into cotton lipid metabolism related genes to harness cottonseed oil's full spectrum of uses more effectively.

Triacylglycerols (TAGs) represent the predominant mechanism for the sequestration and accumulation of neutral lipids within plant organelles known as oil bodies, a critical component contributing to over half of the seed's mass [4, 5]. Lipases, a class of enzymes ubiquitously distributed across the animal, plant and microbial kingdoms, are endowed with the capacity for both hydrolysis and transesterification, acting upon a diverse array of substrates. While there has been considerable research on microbial lipases, the exploration of plant lipases remains underdeveloped and sparsely reported [6]. The breakdown of TAG within plant cells is predominantly facilitated by a trio of lipases: Sugar Dependent 1 (SDP1), Diacylglycerol Lipase (DGL), and Monoacylglycerol Lipase (MGL). These enzymes orchestrate the liberation of free fatty acids and glycerol, subsequently mobilizing carbon reserves essential for seedling development via the β-oxidation pathway [7, 8]. Monoacylglycerol (MAG) undergoes enzymatic hydrolysis, catalyzed by MAGL, into free fatty acids and glycerol. In a pivotal study conducted in 1992, MAGL was classified within the alpha/beta hydrolase superfamily, characterized by its structural arrangement of α helices and β folds. This division highlighted the two conserved domains of the enzyme: the Gly-X-Ser-X-Gly motif and a catalytic triad of serine, aspartic acid and histidine, which is key to its enzymatic activity [9, 10].

Extensive research on monoacylglycerol lipase (MAGL) has predominantly been conducted in mammalian systems [8], where MAGL is recognized for its role as a pivotal fat mobilizer and it’s influenced on the endogenous cannabinoid signaling pathway through the regulation of intracellular levels of 2-arachidonoylglycerol [11]. Conversely, investigations into MAGL's function within the plant kingdom are notably less extensive, with only a handful of studies focusing on species such as *Arabidopsis thaliana* [6], *Brassica napus * [12], and *Arachis hypogaea* [13]. Notably, in *Arabidopsis*, a subset of sixteen genes has been identified within the *MAGL* gene family, with a significant majority (11 out of 16) demonstrating the capability to encode functional MAGL enzymes [6]. Among these, *AtMAGL6* and *AtMAGL8* are distinguished by their robust hydrolytic activities, playing critical roles in the mobilization of plastidial and extraplastidial membrane lipids during the process of leaf senescence [14]. Furthermore, *AtMAGL3*, initially identified as the lysoPL2 enzyme, exhibits both His-X_4_-Asp acyltransferase and Gly-X-Ser-X-Gly motif, encompassing MGAT and acyl hydrolase activities [15], and is thought to function as a caffeyl shikimate esterase involved in lignin biosynthesis [16]. Interestingly, *AtMAGL3* expression is widespread across various organs, with notably higher expression observed in roots and stems [6], suggesting its involvement in a broad spectrum of biological processes. In *Arachis hypogaea*, a total of twenty-four *MAGL* genes have been identified, with *AhMAGL1a/b* and *AhMAGL3a/b* functioning as both hydrolases and acyltransferases. The overexpression of these genes in peanut has been shown to decrease seed oil content and alter fatty acid composition, indicating their potential role in TAG hydrolysis [13]. Similarly, in *Brassica napus*, forty-seven MAGL members have been identified, with the heterologous expression of *BnaC.MAGL8.a* in the microspore wall layer inducing aberrant microsporogenesis and contributing to the onset of male sterility in *Arabidopsis* [12].

In current studies, our investigations reveal *GhMAGL* gene family members in upland cotton from multiple approach unveil a comprehensive analysis encompassing physical and chemical properties, phylogenetic relationships, gene structures, and chromosomal distributions. Subsequently, these genes were analyzed at the genome-wide level has identified elite haplotypes associated with important agronomic traits. Through the elucidation of the functional implications of the *GhMAGL* gene family, our research provides valuable genetic markers for the targeted improvement of cotton, particularly in traits crucial for early maturity, yield optimization, and the enhancement of seed nutritional quality.

## Results

### Identification of the *GhMAGL* family members

Following the elimination of redundant sequences, a set of 30 potential *GhMAGL* gene sequences were obtained, each assigned a unique designation ranging from *GhMAGL1* to *GhMAGL30* in accordance with their respective physical locations. The physicochemical properties of all *GhMAGL* genes were displayed in Additional File 1: Table S1. Within this diverse gene set, the amino acid length span from 253 aa (GhMAGL16) to 453 aa (GhMAGL11 and GhMAGL26), and the molecular weights (MW) is fluctuated from 28585.02 Da (GhMAGL16) to 50104.79 Da (GhMAGL26). Moreover, the theoretical isoelectric points (pIs) of these proteins range markedly from 5.73 (GhMAGL19) to 9.37 (GhMAGL30), indicating a broad spectrum of biochemical diversity. Notably, the GhMAGL proteins predominantly exhibit hydrophilic characteristics, with the exception of GhMAGL25. In terms of subcellular localization, a significant fraction of the GhMAGL proteins (14 out of 30) were predicted to reside within the cytoskeleton, suggesting their potential involvement in cellular structure and dynamics. Additionally, six GhMAGL proteins, GhMAGL2, GhMAGL4, GhMAGL5, GhMAGL15, GhMAGL17 and GhMAGL30, were localized in the chloroplast, implicating their probable role in lipid metabolism processes pertinent to photosynthesis. Four *GhMAGL* genes were associated with peroxisomal localization, and three in the plasma membrane, highlighting their possible roles in lipid catabolism and signaling pathways. A select few *GhMAGL*s were also discerned to localize within the cytoskeleton and nucleus, underscoring the multifaceted roles these enzymes may play in cellular physiology and regulatory mechanisms.

### Multiple sequence alignment and phylogenetic analysis of GhMAGL proteins

The comparative analysis of the thirty GhMAGL proteins unveiled a significant degree of sequence conservation, particularly within the G-X-S-X-G structural motifs and the catalytic triad comprising serine, aspartic acid, and histidine residues. Notably, deviations from the canonical G-X-S-X-G motif were observed in GhMAGL4, GhMAGL16, and GhMAGL19, with the initial glycine residue being substituted by aspartic acid in GhMAGL16 (resulting in a D-X-S-X-G motif) and by serine in both GhMAGL4 and GhMAGL19 (yielding S-X-S-X-G motifs). Structural comparisons of these variant proteins with GhMAGL6, which retains the prototypical G-S-X-S-G motif [17] (Additional File 2: Fig. S1), revealed the absence of a U-shaped fold in GhMAGL16 and GhMAGL19 (Additional File 2: Fig. S2). These structural alterations potentially underpin functional and enzymatic activity shifts in the affected genes. Previous reports indicated that analogous structural modifications in *Arabidopsis thaliana* homologs of *GhMAGL19*, specifically *AtMAGL14* and *AtMAGL16*, have been previously correlated with diminished MAGL activity [6].

**Fig. 1:**
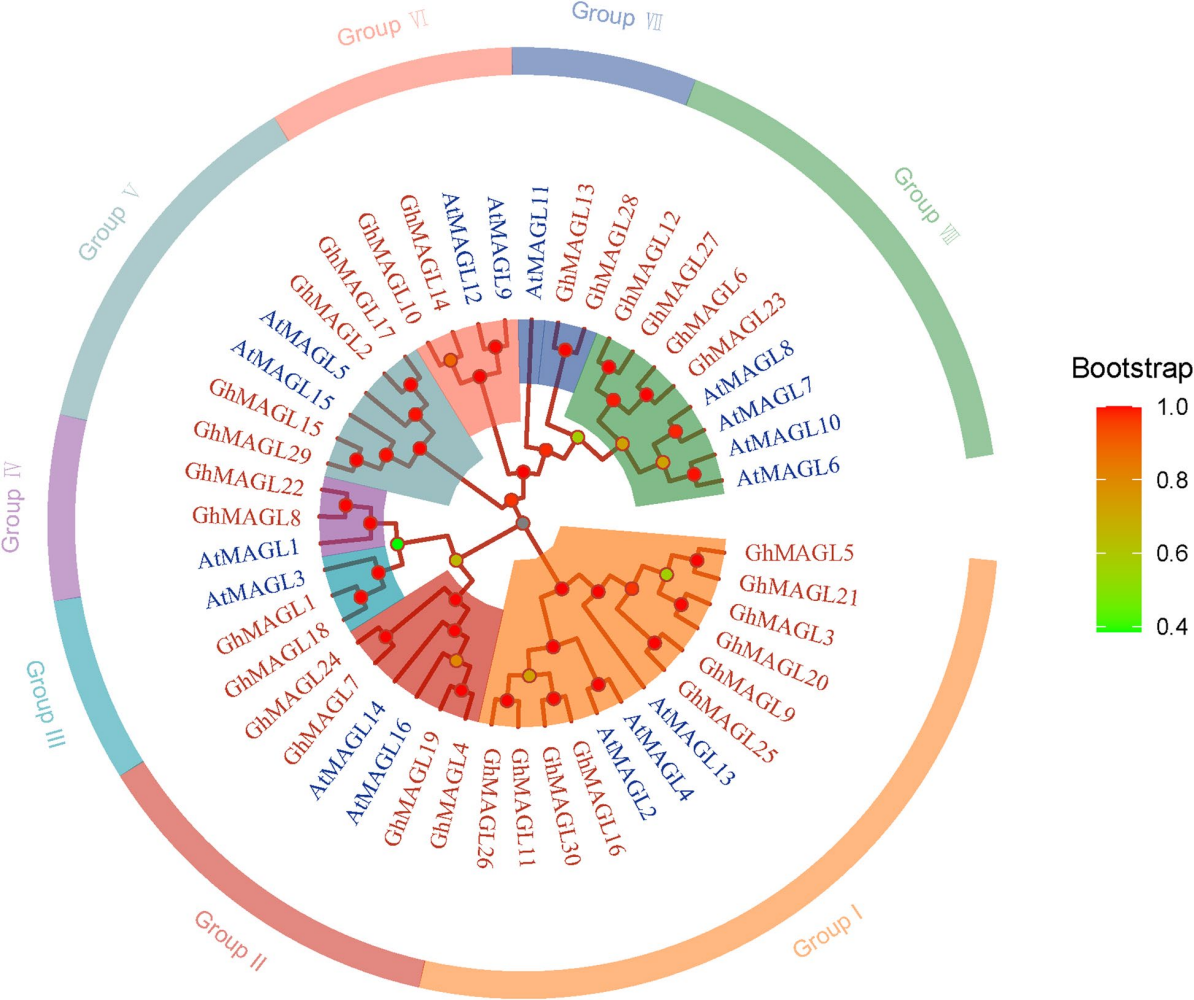
Phylogenetic tree of *MAGL* genes of upland cotton and *Arabidopsis thaliana*, the red font represents *GhMAGL*s and the blue font represents *AtMAGL*s

The phylogenetic investigation based on the amino acid sequences of 30 GhMAGL and 16 AtMAGL proteins facilitated the construction of an evolutionary tree, elucidating the phylogenetic relationships between *MAGL* genes in cotton and *Arabidopsis thaliana* [18]. This evolutionary tree partitioned the 46 MAGL members into 8 distinct phylogenetic subgroups (Fig. 1), corroborating the classifications previously established [6]. Notably, this phylogenetic framework revealed variations in subgroup compositions, highlighted by disparities in gene distribution among these subgroups. Subgroup I emerged as the most members, encompassing 13 genes, while subgroup VIII comprised eight members. Conversely, subgroups III, IV, and VII were identified as the least members, each containing only three members. A comparative analysis of conserved motifs within these homologous genes underscored a shared motif composition, indicative of functional and structural similarities. Additionally, our findings revealed that nine genes: *GhMAGL1*, *GhMAGL2*, *GhMAGL7*, *GhMAGL10*, *GhMAGL14*, *GhMAGL15*, *GhMAGL17*, *GhMAGL24*, and *GhMAGL29* harbored the VX3HGY motif, while *GhMAGL1*, *GhMAGL7*, *GhMAGL8*, *GhMAGL18*, *GhMAGL22*, and *GhMAGL24* contained the His-X_4_-Asp motif, suggesting a substrate specificity and catalytic potential rooted in these conserved sequences. Remarkably, *GhMAGL1*, *GhMAGL7*, and *GhMAGL24* were identified as the sole bifunctional enzymes within the *GhMAGL* gene family, further underlining the diversity of enzymatic roles these proteins play. Intriguingly, all members of subgroups III and IV harbored the His-X_4_-Asp motif, whereas every member of subgroups V and VI exhibited the VX3HGY motif, underscoring a correlation between phylogenetic placement and motif composition.

### Investigation of gene structure and conserved motifs of *MAGL*s in upland cotton

**Fig. 2:**
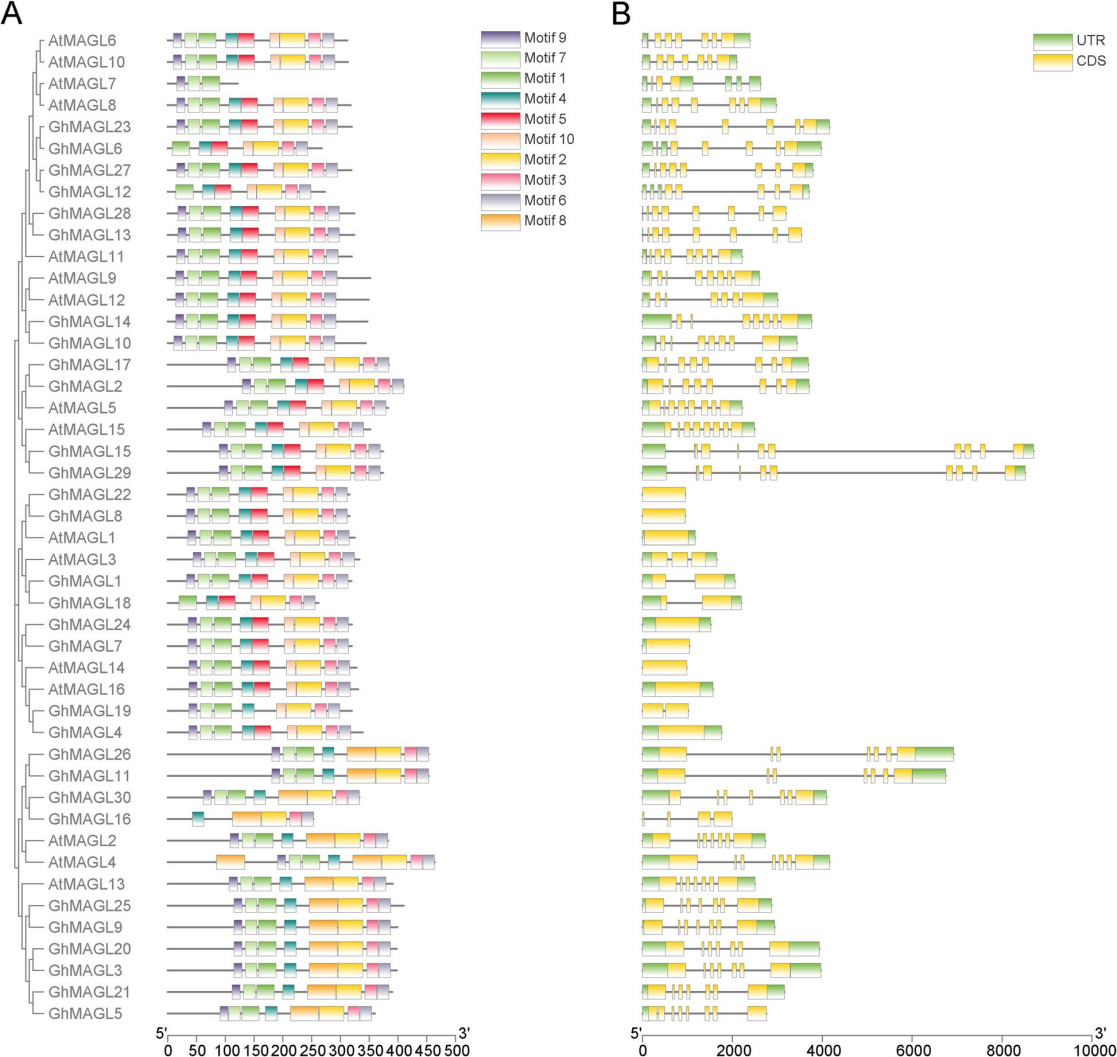
Comparative analysis of conserved motifs and gene structures in the *MAGL* gene family across upland cotton and *Arabidopsis thaliana*. **A** Predicted motifs aligned with the phylogenetic tree of *MAGL* genes, ten conserved motifs are shown in different colored boxes. **B** Gene structure of *GhMAGL*s, exons are depicted by orange boxes, introns by black lines, and the upstream/downstream regions of *GhMAGL* genes are shown as green boxes

To gain more in-depth perspective into the evolution and diversification of *MAGL* gene family in upland cotton, we conducted a comprehensive analysis of their conserved motifs and gene structures (Fig. 2). The distribution of motifs across the subgroups underscores distinct evolutionary pathways, lending further support to the phylogenetic classification of these subgroups (Fig. 2A). *GhMAGL*s within the same subgroup show a remarkable similarity in their conserved motif composition and gene structures, with motifs 4 and 1, identified as integral to the MAGL domain (Additional File 2: Fig. S3), being particularly prevalent. Moreover, motif 5 and motif 10 were universally present across all subgroups, with the notable exception of subgroup I. Conversely, motif 8 was exclusively found within subgroup I, indicating its unique evolutionary trajectory. Furthermore, our analysis revealed substantial structural diversity among the *GhMAGL* genes across different subgroups, manifested in the variation of exon-intron numbers and lengths (Fig. 2B). The exon count varied from 1 to 9, while the number of introns ranged from 0 to 9, highlighting the genetic complexity of the *GhMAGL* gene family. Notably, subgroup V exhibited the greatest complexity in terms of exon-intron structure, with *GhMAGL15* and *GhMAGL29* as representative members. In contrast, genes within subgroups II and IV, except for *GhMAGL19*, were characterized by an absence of introns. It was found that the synteny gene pair *GhMAGL28* and *GhMAGL29*, showcasing a high degree of similarity in both motif composition and exon-intron structure, underlining their close evolutionary relationship. A notable variation in gene length arises from differences in exon-intron lengths, as evidenced by the contrasting sizes of 3,792 bp and 3,191 bp. In conclusion, the different motifs and gene structures between subgroups may be the potential reason of the different functions of the *GhMAGL* gene family.

### Chromosomal localization and synteny analysis of* GhMAGL*s

**Fig. 3:**
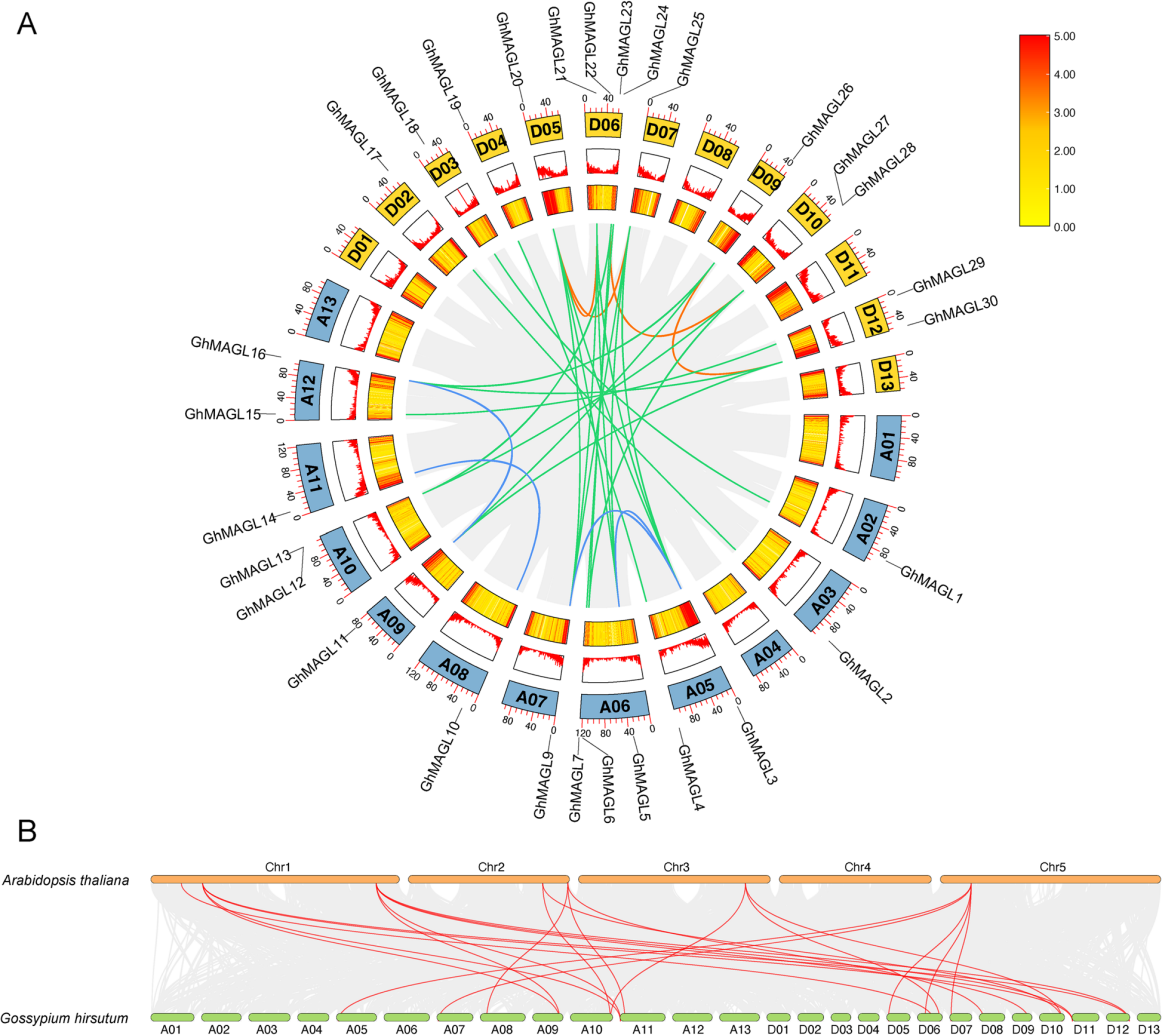
Gene duplication relationship among the *MAGL* genes. **A** Gene duplication relationship among the *MAGL* genes of upland cotton; Blue rectangle represents At subgenome, yellow rectangle represents Dt subgenome; Both line and heat maps represent gene density. Various colors are used to represent different regions of intra-genomic synteny; **B** *MAGL* gene relationship across upland cotton and *Arabidopsis thaliana;* The figure shows the synteny blocks with cotton represented by the gray background, with the *MAGL* gene pairs highlighted by red lines

Utilizing gene physical positions, we depicted the chromosomal mapping of *GhMAGL* genes (Fig. 3A). The 30 identified *GhMAGL* genes were found to be dispersed across 19 chromosomes, except for *GhMAGL8 on* a scaffold. Chromosome D06 harbored the highest number of *GhMAGL* genes (4). Notably, the spatial arrangement of *GhMAGL* genes in upland cotton closely parallels the distribution observed within the *MAGL* gene family in peanuts, predominantly localized at the termini of chromosomes, while a minority occupies central chromosomal regions [13]. Gene duplication events are pivotal in driving the evolutionary diversification of gene families [19]. Our analysis revealed 30 instances of fragment duplication (Fig. 3A, Additional File 1: Table S2), with the majority occurring between the At and Dt subgenomes (21), followed by duplications within the At (4) and Dt subgenomes (4) respectively. Tandem duplications, which often lead to gene conversion and increased sequence homology, play a crucial role in balancing gene family numbers and preserving their functional integrity. Notably, we identified tandem duplication pairs on chromosomes A10 (*GhMAGL12* and *GhMAGL13*) and D10 (*GhMAGL27* and *GhMAGL28*). Thus, fragment duplication emerges as a principal evolutionary force within the *MAGL* gene family.

Furthermore, synteny analysis between upland cotton and *Arabidopsis thaliana* concerning *MAGL* genes identified eighteen homologous gene pairs (Fig. 3B). Remarkably, *GhMAGL12* on chromosome A10 and *GhMAGL27* on chromosome D10 exhibit synteny with *AtMAGL6* in *Arabidopsis thaliana*, aligning with previous findings that *AtMAGL6* and *AtMAGL8* exhibit the highest MAG hydrolase activity [6]. To gain a better understanding of the evolutionary dynamics of *MAGL* genes, the nonsynonymous to synonymous substitution ratio (Ka/Ks) was calculated (Additional File 1: Table S3). Consistently, the duplicated gene pairs demonstrated Ka/Ks values below 1, indicative of purifying selection and highlighting the high sequence conservation within the *GhMAGL* gene family. This observation suggests that the preservation of functional integrity in *GhMAGL*s has likely been facilitated by purifying selection, further contributing to the evolutionary resilience of this gene family.

### Cis-acting elements in promoter regions of *GhMAGL* genes

**Fig. 4:**
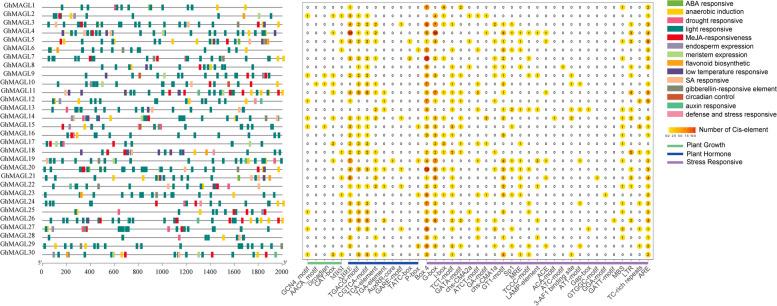
Statistics and categorization of cis-acting elements within the promoter’s region of *GhMAGL*s; Elements with analogous regulatory functions are color-coded together, with the respective quantities of each type displayed on the circle

The promoter regions of genes contain critical cis-acting elements that play a pivotal role in the initiation of transcription by facilitating the binding of transcription factors, thus exerting a profound influence on the regulation of gene expression. In our investigation, an exhaustive analysis identified a total of 779 cis-acting elements across the studied genes (Fig. 4 and Additional File 1: Table S4). Remarkably, the *GhMAGL*s predominantly exhibited the presence of the CAT-box cis-acting element, with 11 instances, which is associated with plant growth and prominently active in meristematic tissues. Additionally, a significant presence of phytohormone-responsive cis-acting elements was observed; notably, 25 *GhMAGL* genes harbored the ABRE element, signaling responsiveness to abscisic acid (ABA), while 22 genes contained the MeJA-responsive element, implicating their potential involvement in jasmonic acid (JA) signaling pathways. These observations suggest that a considerable fraction of *GhMAGLs* may play roles in ABA- and JA-mediated physiological responses. Moreover, our analysis unveiled stress-responsive elements pertinent to light response, low temperature tolerance, anaerobiosis, and drought resistance. Intriguingly, all *GhMAGL* genes were found to possess light-responsive elements such as Box 4 and G-box, indicating their potential involvement in photoperiod-related processes.

### Tissue-specific expression patterns of *GhMAGL* genes

Expression analysis of *GhMAGL*s offer valuable insights into their biological functions. The transcriptome analysis revealed widespread detection of *GhMAGL* genes expression across various plant tissues and different growth stages of TM-1 (Additional File 1: Table S5 and Additional File 2: Fig. S4). Furthermore, a subset of seven *GhMAGL* genes, *GhMAGL1*, *GhMAGL4*, *GhMAGL5*, *GhMAGL9*, *GhMAGL18*, *GhMAGL22*, and *GhMAGL29*, demonstrated significant differential expression between ovule and fiber tissues during the 20-25 DPA period. This differential expression pattern suggests a pivotal role of these genes in regulating nutrient mobilization during the early stages of cottonseed development. Notably, genes classified within the same phylogenetic subgroup tended to exhibit concordant expression profiles, underscoring the likelihood of shared regulatory mechanisms or functional similarities. For example, *GhMAGL2* and *GhMAGL17* showed predominant expression in the pistil and were minimally expressed in other tissues, hinting at their specific involvement in floral development and reproductive success. In summary, the comprehensive expression analysis of *GhMAGL* genes throughout the reproductive cycle of cotton delineates their integral contribution to various aspects of plant growth and development. These findings not only advance our understanding of the regulatory and functional diversity within the *GhMAGL* gene family but also highlight the potential of these genes as key players in the optimization of cottonseed yield and quality.

### Association and haplotype analysis of *MAGL*s in upland cotton

**Fig. 5:**
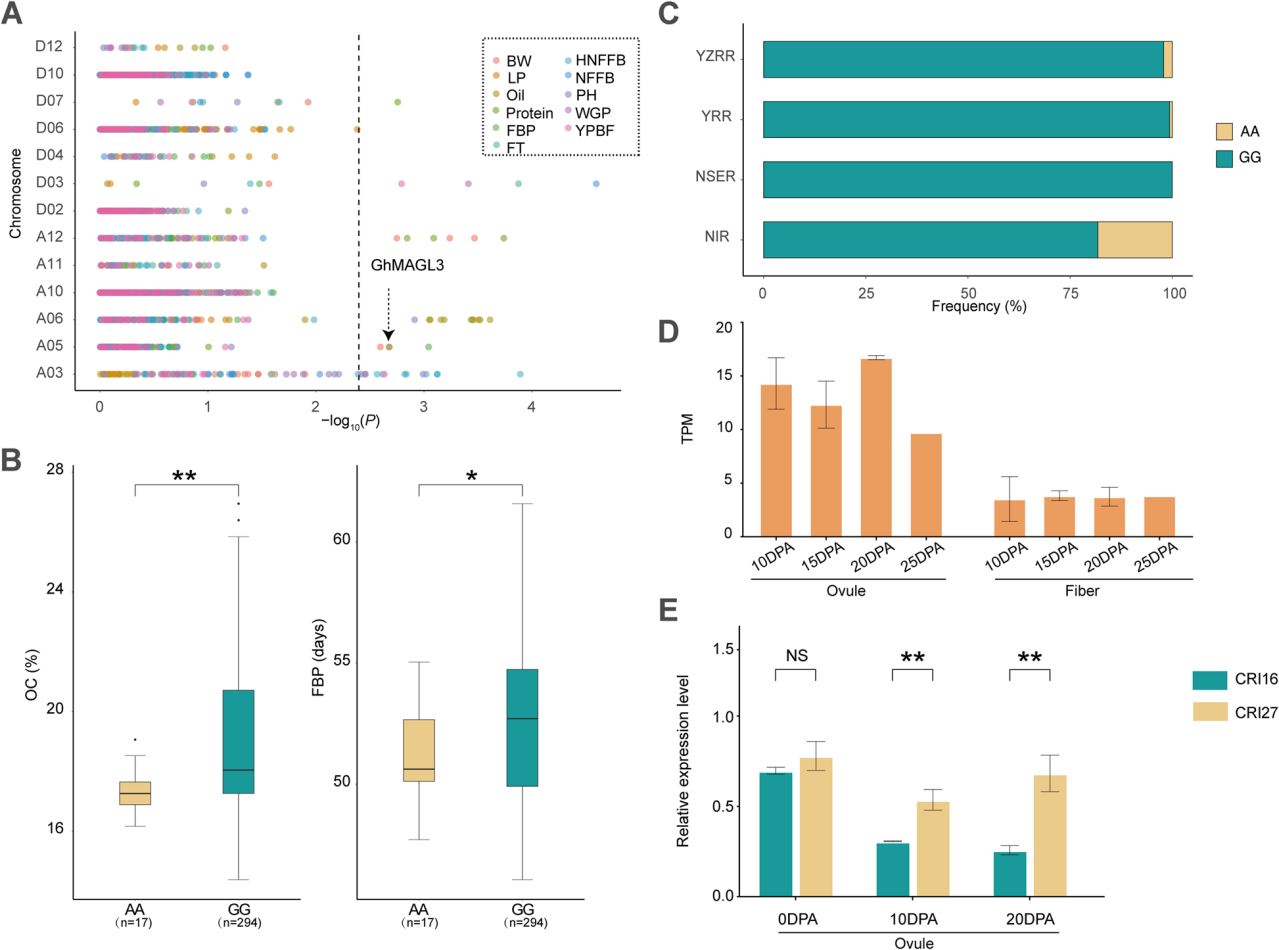
Variation analysis of oil content (OC) related trait associated with *GhMAGL3*. **A** Manhattan plots for association mapping between 11 traits of cotton; **B** Box plots for OC and FBP of the two haplotypes mentioned above; **C** The haplotype distribution of *GhMAGL3* varies across four cotton plant regions; **D** Tissue specific analysis of *GhMAGL3* in ovules and fibers at 10-25 DPA; (**E**) qRT-PCR analysis of *GhMAGL3*, 'CRI16' is high oil content accession, 'CRI27' is low oil content accession. The significance is shown below: **, * and NS represent *P*<0.01, *P*<0.05, *P*>0.05, respectively

Association analyses were conducted employing GEMMA software with a mixed linear model (MLM), utilizing both phenotypic and genotypic data from 355 accessions to investigate associations within 30 members of the *GhMAGL* gene family (Additional File 1: Table S6). A comprehensive analysis identified 236 SNPs located within 2 kb upstream and downstream of the *GhMAGLs*. These SNPs were subsequently analyzed for association with 11 phenotypic traits (Additional File 1: Table S7). Notably, the SNPs significantly associated with these traits were predominantly located within exons, 5'UTRs, and 3'UTRs regions of the genes. Among the associated *GhMAGL* genes, *GhMAGL3* and *GhMAGL18* emerged as the genes associated to the greatest number of traits (four each), underscoring their potential as stable genes involved in various phenotypic expressions. *GhMAGL3*, in particular, demonstrated a linkage to oil content via GWAS and is a homolog to *AtMAGL13 (*Fig. 5A*)*, which located in the endoplasmic reticulum (ER) involved in acyl lipid metabolism. Prior research indicates that *AtMAGL13* is upregulated in the epidermis of upper stems, suggesting its role in the efficient degradation of TAGs and/or remodeling of membrane lipids [20]. This leads us to hypothesize a similar function for *GhMAGL3*. Haplotype analysis across 355 cotton accessions revealed two dominant haplotypes for *GhMAGL3*: Hap1 (AA), associated with lower oil content, and Hap2 (GG), linked to higher oil content (Fig. 5B and Additional File 1: Table S8). To further explore the genetic basis of these haplotypes and their association with geographical distribution, we categorized the 355 upland cotton varieties into four regional groups (Northwest Inland region: NIR, Northern Specific Early maturity region: NSER, Yellow River region: YRR and Yangzi River region: YZRR). Hap1 (AA) was found to be predominant in the NIR (Fig. 5C). Public transcriptome data analysis has shown that *GhMAGL3* exhibits higher expression in ovules than in fibers during the critical phase of oil accumulation (Fig. 5D). Further, during seed development, the expression level of *GhMAGL3* in low oil content accession (‘CRI27’) is surpassed that in the high oil content accession (‘CRI16’) (Fig. 5E), suggesting a negative regulatory effect. Additionally, varieties harboring the GG haplotype displayed an extended FBP (Fig. 5B), suggesting enhanced conditions for sustained oil synthesis and accumulation.

**Fig. 6:**
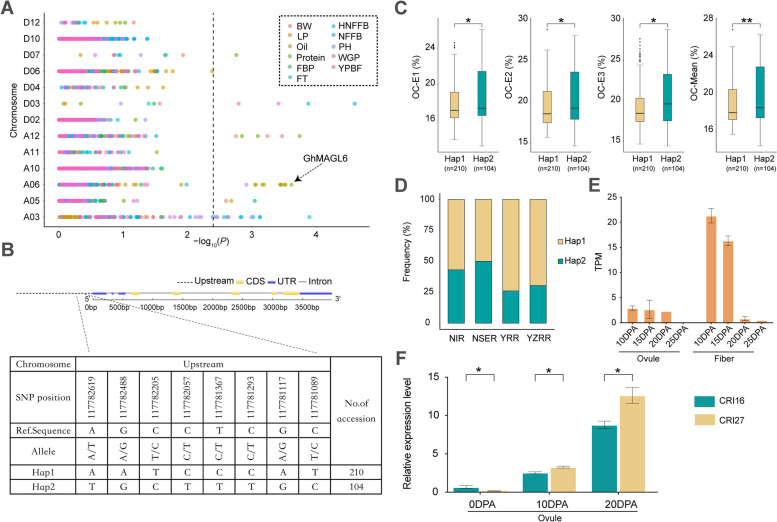
Variation analysis of oil content (OC) related trait associated with *GhMAGL6*. **A** Manhattan plots for association mapping between 11 traits of cotton; **B** Gene structure and haplotype analysis of *GhMAGL6*; **C** Boxplot for oil content between different haplotypes. E1, E2, E3 represent the oil content of cottonseed planted in Liaocheng, Shandong, Huanggang, Hubei and Sanya, Hainan in 2021; Mean represents the average of oil content in three environments; **D** The haplotype distribution of *GhMAGL6* varies across four eco-cotton regions; **E** Tissue specific analysis of *GhMAGL6* in ovules and fibers at 10-25 DPA. (**F**) qRT-PCR analysis of *GhMAGL6*. 'CRI16' is high oil content accession, 'CRI27' is low oil content accession. The significance is shown below: ** and * represent *P*<0.01, *P*<0.05, respectively

Another gene associated with oil content regulation is *GhMAGL6*, which resides within the same phylogenetic subgroup as *AtMAGL6* (Fig. 6A). This association is of particular interest given that *AtMAGL6* has been previously confirmed as possessing the strongest hydrolytic activity within its gene family [6]. This similarity suggests that *GhMAGL6* might play a comparable role in lipid metabolism regulation in upland cotton. Our analysis identified seven SNP loci upstream of *GhMAGL6* (Fig. 6B and Additional File 1: Table S8). Two major haplotypes of *GhMAGL6* were delineated, with Hap2 being less frequent than Hap1, but varieties containing Hap2 have higher oil content (Fig. 6C). Geographical distribution analysis of these haplotypes across different cotton-growing regions revealed a lower prevalence of the elite haplotype Hap2 in the YRR and YZRR compared to the NIR and NSER (Fig. 6D). This distribution pattern correlates with previous observations that cottonseed oil content tends to decrease at lower latitudes [21]. According to the results of tissue-specific analysis, *GhMAGL6* is mainly expressed in fibers and ovules (Fig. 6E). Moreover, qRT-PCR analysis demonstrated higher expression levels of *GhMAGL6* in low oil content variety (Fig. 6F), suggesting a negative regulatory effect. The expression of *GhMAGL6* may have led to the hydrolysis of lipids into free fatty acids, which finally promotes fiber growth [22].

## Discussion

### Evolutionary expansion and functional analysis of *GhMAGL* genes in upland cotton

It is well known that hydrolysis catalysis of TAG, which is essential for plant growth cycle, yet while considerable research has been devoted to the initial stages of TAG hydrolysis, MAGL has received comparatively less attention. In this study, we conducted a comprehensive investigation of the *GhMAGL*s, identifying 30 candidate *GhMAGL* genes classified into eight distinct subgroups. The enumeration of *MAGL*s in upland cotton is 1.875 times that of *Arabidopsis thaliana* (16) but less than *Brassica napus* (47) [12] and exceeds the count in cultivated peanut (24) [13]. This variation is primarily attributed to gene duplication through evolutionary processes, with upland cotton is an allotetraploid, arising from the hybridization of two ancestral species, leading to a genome doubling and an increased gene copy number. Synteny analysis of genes of the *GhMAGL* gene family identified 30 synteny relationships, which indicates the expansion of *GhMAGL* gene family during the evolutionary process. Notably, two tandem replication pairs were identified on chromosome A10 (*GhMAGL12* and *GhMAGL13*) and chromosome D10 (*GhMAGL27* and *GhMAGL28*), with segmental duplication emerging as a significant driver of the *GhMAGL* gene family's duplication. Furthermore, 18 synteny relationships were observed between upland cotton and *Arabidopsis thaliana*, suggesting potential functional conservation across these homologous genes. Interestingly, *GhMAGL4*, *GhMAGL16*, *GhMAGL19* were observed to have a change in the first Gly of its G-S-X-S-G conserved domain, and *GhMAGL16* and *GhMAGL19* were not expressed or relatively lower expressed during ovule development stage according to the RNA-seq analysis, inferring that these amino acid substitutions may compromise MAGL activity (Additional File 2: Fig. S5).

### Functional insights and regulatory mechanisms of *GhMAGL* genes in lipid metabolism

Predictive analysis of subcellular localization demonstrated that the majority of the *GhMAGL*s are predominantly situated the cytoplasm, plasma membrane, and chloroplasts, a distribution pattern that is consistent with observations conducted in *Arabidopsis thaliana* [6]. The pronounced presence of GhMAGL proteins on the cell membrane underscores their potential role in the regulation of glycerol-3-phosphate (G-3-P) production, a crucial precursor in lipid biosynthesis, thereby influencing lipid accumulation within the cell. The organization of gene structure and the distribution of conserved motifs offer auxiliary insights into the evolutionary relationships among species or genes [23]. A characteristic arrangement of most conserved motifs, following the sequence 9-7-1-4-2-3-6 (Fig. 2A), is observed, with exceptions noted for *GhMAGL6*, *GhMAGL12*, *GhMAGL16*, and *GhMAGL18*, which lack motif 9. Additionally, motif 8 is exclusively found in subgroup I, while motifs 5 and 10 are present in other subgroups. All members of subgroup III and subgroup IV contained acyltransferase motif (His-X_4_-Asp). And all members of subgroup V and VI contained lipid-binding motifs (VX3HGY). It is striking that they all have similar gene structures, indicating that these genes may have evolved distinct functional roles from other family members. The examination of promoter cis-acting elements revealed that all *GhMAGL* gene family members possess light-responsive elements, highlighting the potential role of light as an environmental cue in modulating photosynthetic carbon fixation and subsequent physiological processes through complex signaling pathways [1]. Previous studies have illustrated how shade conditions can hinder the rapid phase of oil accumulation in tung seed oil [24], whereas exposure to high light intensity can induce metabolic shifts in *Chlorella vulgaris*, leading to increased oil accumulation [25]. This could suggest that the light conditions may influence lipid metabolism gene expression, with potential downregulation in darker conditions impeding lipid biosynthesis in developing embryos and affecting lipid content accumulation [26]. Consequently, the light-responsive cis-acting elements identified in *GhMAGL* promoters are likely to have a profound impact on fatty acid synthesis and degradation pathways, offering new insights into the regulatory mechanisms that regulate lipid accumulation in plants.

### Genetic variants and functional roles of *GhMAGL3* and *GhMAGL6* in cotton lipid metabolism

**Fig. 7:**
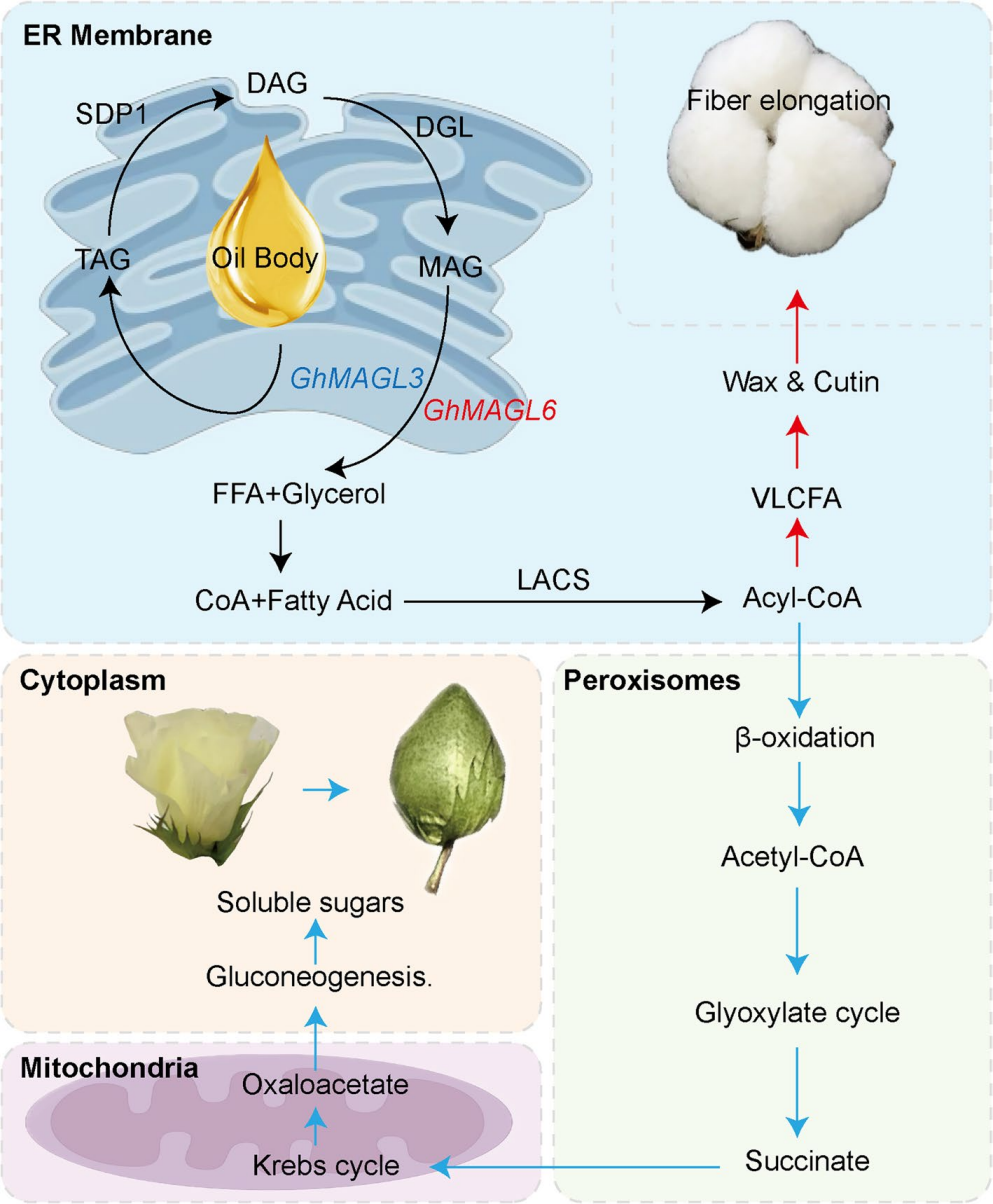
Potential working mechanisms of *GhMAGL3* and *GhMAGL6* in different subcellular compartments (By Figdraw); The blue arrow represents the possible regulatory pathways of *GhMAGL3*, while the red arrow represents the possible regulatory pathways of *GhMAGL6*. Abbreviations: TAG, triacylglycerol; SDP1, Sugar dependant 1; DAG, diacylglycerol; DGL, diacylglycerol lipase; MAG, monoacylglycerol; FFA, free fatty acid; CoA, acetyl-coenzyme A; LACS, long-chain acyl-CoA synthetase; VLCFA, very long chain fatty acid

Oil content is an important quantitative trait that has been studied extensively in various crops. Tang et al. conducted an association analysis on 505 inbred lines of rapeseed and identified a pair of homologous genes, *BnPMT6s,* which negatively regulate seed oil content in two QTLs [27]. Liu and colleagues identified several genes associated with lipid synthesis in soybeans, including *GmPDAT*, *GmAGT*, *GmACP4*, *GmZF351*, and *GmPgs1*, through genome-wide association analysis and multi-omics analysis [28]. Zhang et al. identified the genes *GhACP2*, *GhHSL1*, *GhLEC1*, and *GhFAD2* within stable QTLs through association analysis using a RIL population in cotton [29]. However, there is a lack of research on the role of *MAGL* genes in regulating cotton oil content. Based on the expression levels of *GhMAGL*s, association analysis and haplotype analysis, two genes (*GhMAGL3* and *GhMAGL6*) have been identified as potential candidates for regulating lipid biosynthesis and degradation (Fig. 7). The identification of *GhMAGL3* and *GhMAGL18* as key genes associated with a broad spectrum of traits underscores their potential roles as central regulators within the lipid metabolism pathway, echoing findings in *Arabidopsis thaliana* where homologous genes like *AtMAGL13* have been implicated in similar metabolic processes [20]. Haplotype analysis of *GhMAGL3* revealed a predominance of the Hap1 (AA) haplotype in the Northwest Inland Region (NIR), which encompasses both southern and northern Xinjiang (Fig. 5C). Southern Xinjiang, characterized by its higher altitude and lower latitude, benefits from increased sunlight exposure relative to northern Xinjiang, which, due to its lower altitude and higher latitude, experiences reduced sunlight exposure. Previous studies have reported a significant positive correlation between cottonseed oil content and sunshine hours [30], due to the prolonged sunshine exposure in the southern Xinjiang, cultivation in this region harbors a higher frequency of GG haplotypes associated with enhanced oil content. To elucidate the roles of *GhMAGL3* and *GhMAGL6* in cotton lipid metabolism, we hypothesize a regulatory model based on known lipid degradation and transport pathways. The ER serves as a crucial site for lipid synthesis, with TAG being key constituents of oil bodies. Cellular lipases, including *GhMAGL3* located in the cytoskeleton plasm, initiate TAG breakdown, releasing free fatty acids and glycerol. The metabolic products are then directed to peroxisomes for β-oxidation, leading to succinate formation via the glyoxylate cycle. This succinate is transported to the mitochondria, where it is converted into malate, an essential component of the Krebs cycle. The resulting malate is transported to the cytoplasm, converted into oxaloacetate, and subsequently metabolized through gluconeogenesis to produce soluble sugars that support reproductive plant growth. Thus, we supposed that *GhMAGL3* participated in the activation of gluconeogenesis in cytoskeleton plasm [31]. *GhMAGL6* is detected flavonoid element and have higher expression in fiber development, we speculate reactions by *GhMAGL6* may undergo fatty acid synthase (FAS) and elongation to form saturated fatty acid and acetyl-coenzyme A (CoA). It forms very long chain fatty acid (VLCFA) under the catalysis of long-chain acyl-CoA synthetase (LACS) [7]. VLCFA stimulate fiber elongation by enhancing the production of wax or cutin and the expression of ETH biosynthesis [22]. Hence, it is proposed that *GhMAGL6* may be mainly to participate in the process of lipid hydrolysis to elongate fibers. Additional experiments are warranted to delve deeper into the mechanisms by which *GhMAGL*s catalyze metabolic reactions.

## Conclusions

In brief, these findings elucidate the potential of specific members of the *GhMAGL* gene family as targets for genetic improvement. The correlation between gene expression, haplotype variation, and trait phenotypes offers a promising avenue for the development of molecular markers and breeding strategies aimed at the precise molecular mechanisms by which *GhMAGL* genes influence lipid metabolism and to explore their potential applications in cotton breeding programs.

## Materials and methods

### Identification of *MAGL* genes in upland cotton

Genomic information of *Gossypium hirsutum* L. was obtained from the Cottongen database (https://www.cottongen.org/species/Gossypium_hirsutum/HAU-AD1_genome_v1.0_v1.1) [32]. In parallel, the MAGL protein sequences of *Arabidopsis thaliana* were downloaded from the Arabidopsis Information Resource (TAIR) (https://www.arabidopsis.org/download/). Subsequently, the amino acid sequence of AtMAGLs was analyzed by using the BLAST program, adopting a stringent significance threshold characterized by an E-value of less than 1 × 10^−20^. The confirmation of *MAGL* members was achieved by searching for the presence of G-X-S-X-G conserved domains (PF12146) in the target genes using the Pfam database (http://pfam.xfam.org/) [33] and SMART domain search database (http://smart.embl.de/) [34]. Subsequent investigations delved into the physicochemical attributes of GhMAGL proteins, encompassing analyses of protein length (aa), molecular weights (MW), isoelectric points (pIs), and the Grand average of hydropathicity (GRAVY) indices, utilizing the ProtParam tool (https://web.expasy.org/protparam/). Furthermore, predictive insights into the subcellular localization of these proteins were garnered through the WOLF-PSORT platform (https://wolfpsort.hgc.jp/).

### Phylogenetic analysis of *MAGL* family genes

Multiple sequence alignments were conducted by MEGA software (version: MEGA X) [35] and visualized with GeneDoc (version: 2.7) [36]. Structure modeling of MAGL proteins in upland cotton was generated through the Phyre website (version: 2.0) (http://www.sbg.bio.ic.ac.uk/phyre2) and visualized by PyMoL software (version: 2.5.8). Subsequently, the Maximum Likelihood (ML) tree was built under 1,000 bootstrap replicates [37] and optimal LG and G+I (Gamma Distributed with Invariant Sites) as best model. Finally, the visualization was created using the 'ggtree' package in the R software (version: 4.3.2) [38]. The classification of *GhMAGL*s were referred from the previous study in *Arabidopsis thaliana* [6]*.*

### Gene structure and conserved motif analysis of *GhMAGL*s

The gff3 files of the *MAGL* genes from the *Gossypium hirsutum* genome (version: HAU-AD1) were submitted to the GSDS online tool (version: 2.0) (http://gsds.gao-lab.org/) to display their gene structures [39]. The MEME website (version 5.5.1) (https://meme-suite.org/meme/tools/meme) was used to identify the conserved motifs of the *MAGL* genes [40]. The MEME analysis was performed using specific parameters, including an extensive search for motifs with no limitation on the number of repetitions, a maximum allowance of 10 identified motifs, and default settings for other analysis parameters. Visualization was drawn by TBtools software (version: 2.034) [41].

### Chromosomal location and synteny analysis of *GhMAGL*s

The chromosomal distribution of the *MAGL* genes and their synteny relationships were investigated using the One Step MCScanX function and visualized through the Advanced Circos feature of the TBtools software (version: 2.034) [41]. The Simple Ka/Ks Calculator tool in TBtools software (version: 2.034) was employed to compute the non-synonymous/synonymous (Ka/Ks) ratios.

### Cis-acting elements analysis

Promoter sequences (2,000 bp in upstream region) of *GhMAGL* genes were downloaded from the cotton genome database (https://www.cottongen.org/species/Gossypium_hirsutum/HAU-AD1_genome_v1.0_v1.1) [32]. The subsequent prediction of cis-acting regulatory elements within these sequences was conducted using the PlantCARE database (http://bioinformatics.psb.ugent.be/webtools/plantcare/html/) [42], and visualized utilizing the TBtools software (version: 2.034).

### Expression profiling of *GhMAGL* genes by RNA-seq

Public transcriptome data of 'TM-1' was consistent with previous study [43], it was obtained from the SRA module of NCBI (https://www.ncbi.nlm.nih.gov/sra/)(database number: PRJNA490626) [44], 30 *GhMAGL*s were selected for expression analysis across various tissues (root, stem, leaf, torus, sepal, bract, filament, pistil, anther) and developmental stages (ovule and fiber at 10 to 25 day post-anthesis). The transcriptome data were processed using the salmon software (version: 0.13.1) to quantify gene expression levels [45], which were then normalized to TPM values. The normalization formula accounted for the read count and exon length of each gene, ensuring an accurate representation of gene expression levels across samples. The normalized data was log-transformed (log_2_(x+0.01)) and visualized through the 'pheatmap' R-package [46].

### qRT-PCR analysis of *GhMAGL* genes

Accessions with contrasting oil content levels, including high oil varieties ('CRI16') and low oil varieties ('CRI27'), were cultivated in Hangzhou, Zhejiang, China (30.23˚ N, 117.93˚ E) in 2023. The experimental design employed a randomized block design. Ovule samples were collected at 0, 10, 20 DPA, the bolls were rapidly frozen in liquid nitrogen, the ovules were carefully extracted and stored at -80°C for subsequent experiments. Total RNA extraction from cotton ovule was carried out using the RNAprep Pure Plant Kit from Vazyme (Nanjing, China). Subsequently, 20 µl cDNA was synthesized through reverse transcription of the extracted total RNA using the HiScript II QRT SuperMix for qPCR (+gDNA wiper). To facilitate gene expression analysis primers were designed by SnapGene software (version: 6.0.2) (https://www.snapgene.com/) (Additional File 1: Table S9). The quantitative real-time PCR (qRT-PCR) experiments were performed on LightCycler 480 II PCR System (Mannheim, Germany), with *GhActin* selected as the internal reference gene for normalization purposes [47]. Quantification of gene transcript levels was performed using the 2^-∆∆CT^ method with three biological replicates and three technical replicates [48]. The expression data was visualized by GraphPad Prism software (version: 10) and the 'pheatmap' R-package to generate comprehensive and informative representations of gene expression patterns within the studied gene set [46].

### Association analysis and Haplotype analysis of *GhMAGL* genes with important agronomic traits in upland cotton

Resequencing of the natural population libraries was conducted using the Illumina HiSeq 4000 platform, producing 150 bp paired-end reads, as previously reported [49]. To mine elite SNPs associated with *GhMAGL*s, as well as the impact on cotton traits (Boll weight: BW, Lint percent: LP, Oil content: OC, Protein content: PC, Period from the first flower blooming to the first boll opening: FBP, Flower time: FT, Height of the node of the first fruiting branch: HNFFB, Node of the first fruiting branch: NFFB, Plant height: PH, Whole growth period: WGP, Yield percentage before frost: YPBF) [49–51]. HAU_v1.1 has been selected as a reference for the GWAS. A total of 236 SNPs were identified within 2 kb upstream and downstream of 30 *MAGL* genes, each demonstrating a minor allele frequency (MAF) exceeding 0.05 and a missing rate below 20% across 355 sequenced cotton accessions [52]. Then we used an MLM model with Gemma (version: 0.98.5) to perform association analysis on the corresponding SNP data of *GhMAGL* genes [53]. The threshold was calculated using the Bonferroni correction, resulting in a value of 2.37 (-log_10_^(1/236)^, where 236 represents the total number of SNPs) [54]. Additionally, haplotypes of the *GhMAGL* genes were determined using Haploview software [55] and identified within 355 cotton germplasm accessions. These haplotypes were analyzed for their distribution across domestic cotton cultivation regions, aligning with classifications of breeding stages and geographical distributions previously characterized by our laboratory [56]. The visualization of these comprehensive analyses was performed using the 'ggplot2' package within R software (version: 4.3.2) [57].

### Supplementary Information

Supplementary Material 1. 

Supplementary Material 2. 

## Data Availability

The related gene sequence files of all cotton were downloaded from CottonGen (https://www.cottongen.org/). *Arabidopsis thaliana* was downloaded from TAIR (https://www.arabidopsis.org/). The public transcriptome data were downloaded from the SRA module of NCBI (https://www.ncbi.nlm.nih.gov/sra/)(database number: PRJNA490626). The resequencing data were downloaded from the Bioproject module of NCBI (https://www.ncbi.nlm.nih.gov/bioproject/) (database number: PRJNA389777).
