# Supplementary material for: Genome-wide identification and mining elite allele variation of the Monoacylglycerol lipase (MAGL) gene family in upland cotton (Gossypium hirsutum L.)
Source: BMC Plant Biol. 2024 Jun 21;24:587. doi: 10.1186/s12870-024-05297-w (PMC11191281; doi:10.1186/s12870-024-05297-w)
Supplement: Supplementary file 2 — Supplementary Material 2. [file 12870_2024_5297_MOESM2_ESM.docx]

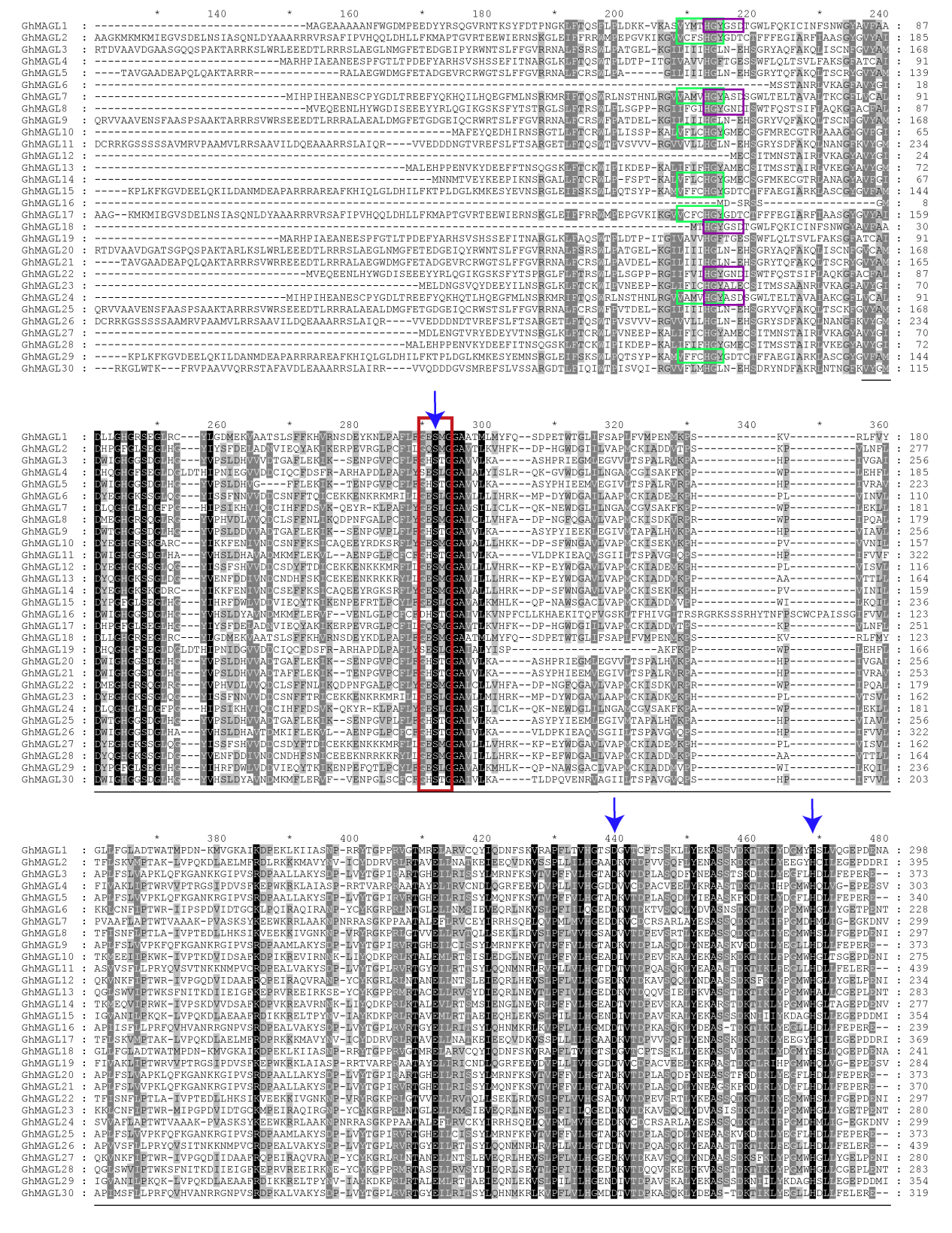


**Figure S1:** Sequence alignment of 30 *GhMAGL* genes. Shades of gray or black are used to indicate conservative or identical amino acids. The alpha/beta hydrolase conserved domain was underscored, the conservative regions of G-X-S-X-G, VX3HGY and His-X_4_-Asp framed boxed in red, green, and purple, respectively. The blue arrows highlight the catalytic triad (serine, aspartic acid and histidine).


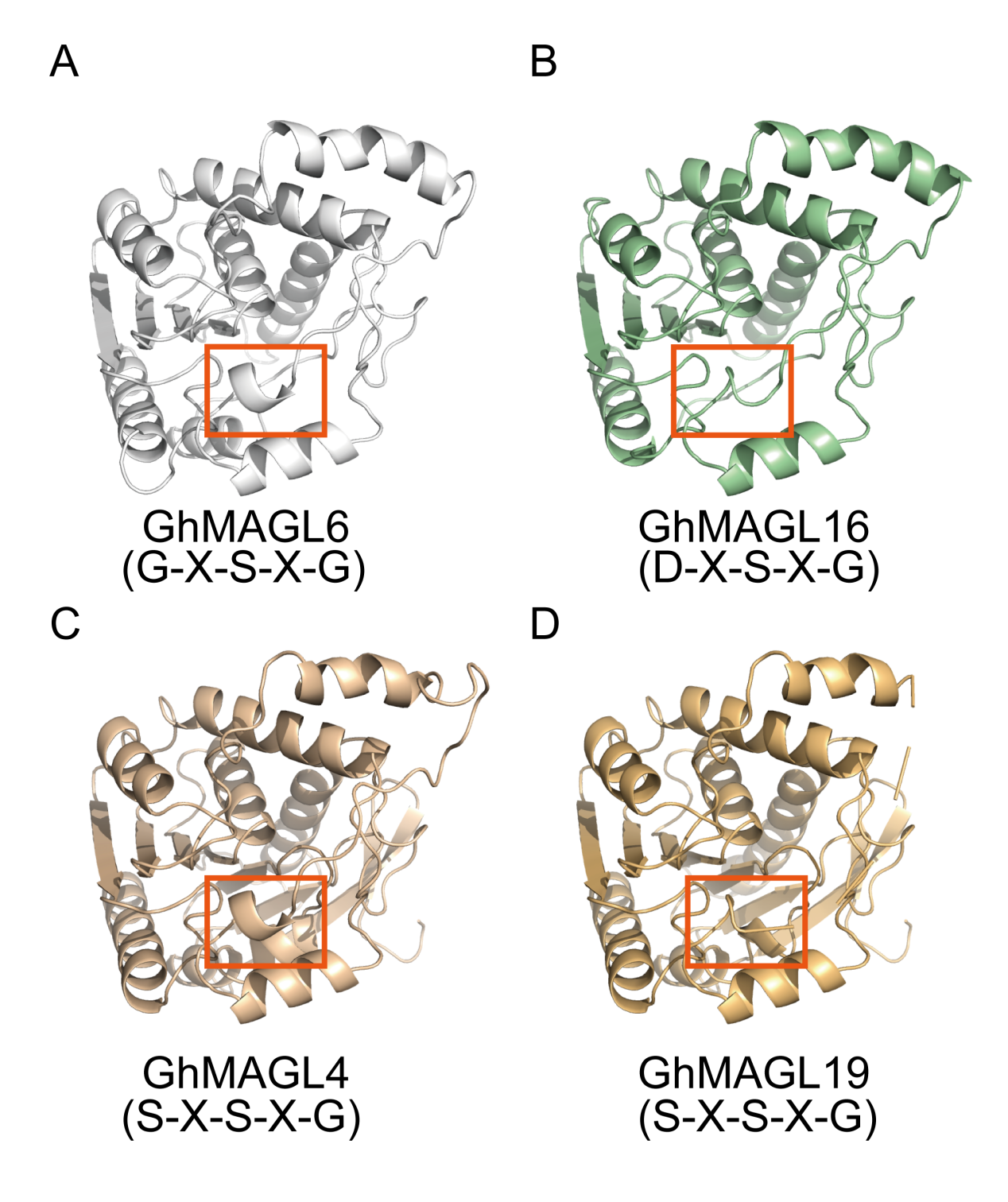


**Figure S2:** Comparison of three-dimensional protein models. (A) *GhMAGL6* contains complete G-X-S-X-G motif, (B) *GhMAGL16* contains D-X-S-X-G motif, (C) *GhMAGL4* contains S-X-S-X-G motif. (D) *GhMAGL19* contains S-X-S-X-G motif. Highlight the structural differences between the four genes with orange box.


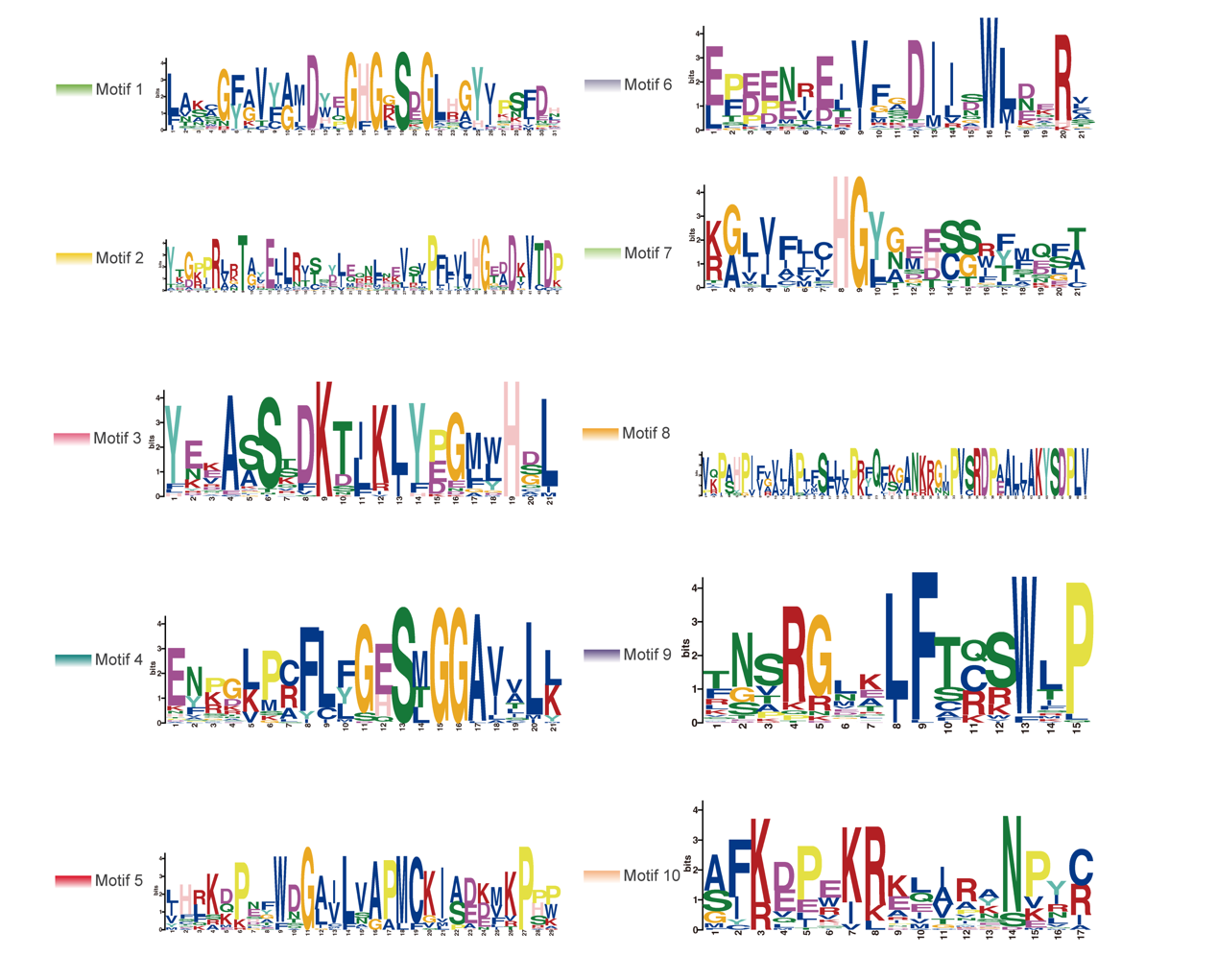


**Figure S3:** Detailed sequences of the predicted MAGL motif


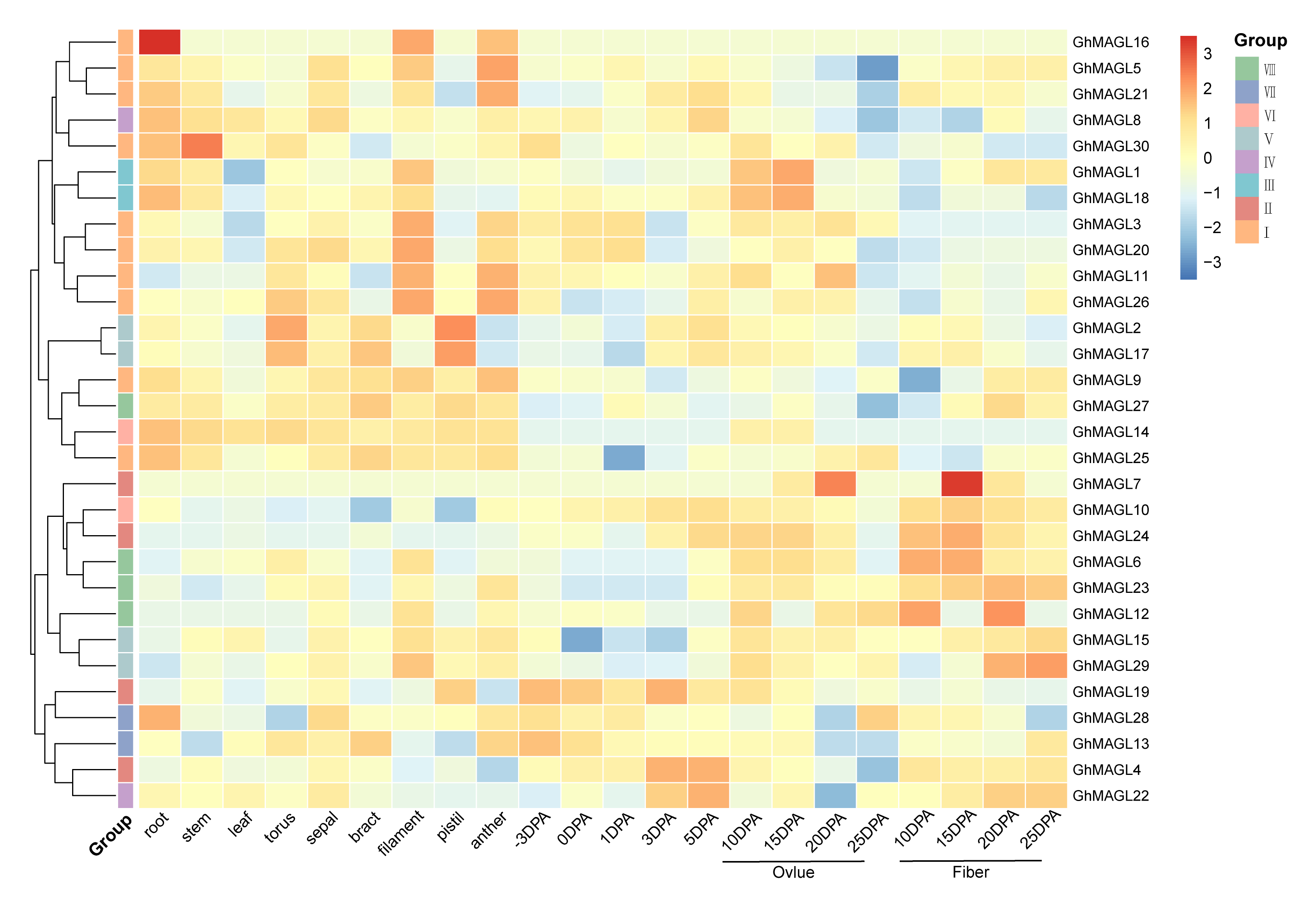


**Figure S4:** The tissue-specific expression patterns of *GhMAGL*s. The heatmap of tissue-specific expression patterns in 30 *GhMAGL*s. DPA, days post anthesis. The bottom bar indicates low to high normalized expression data (blue to red).


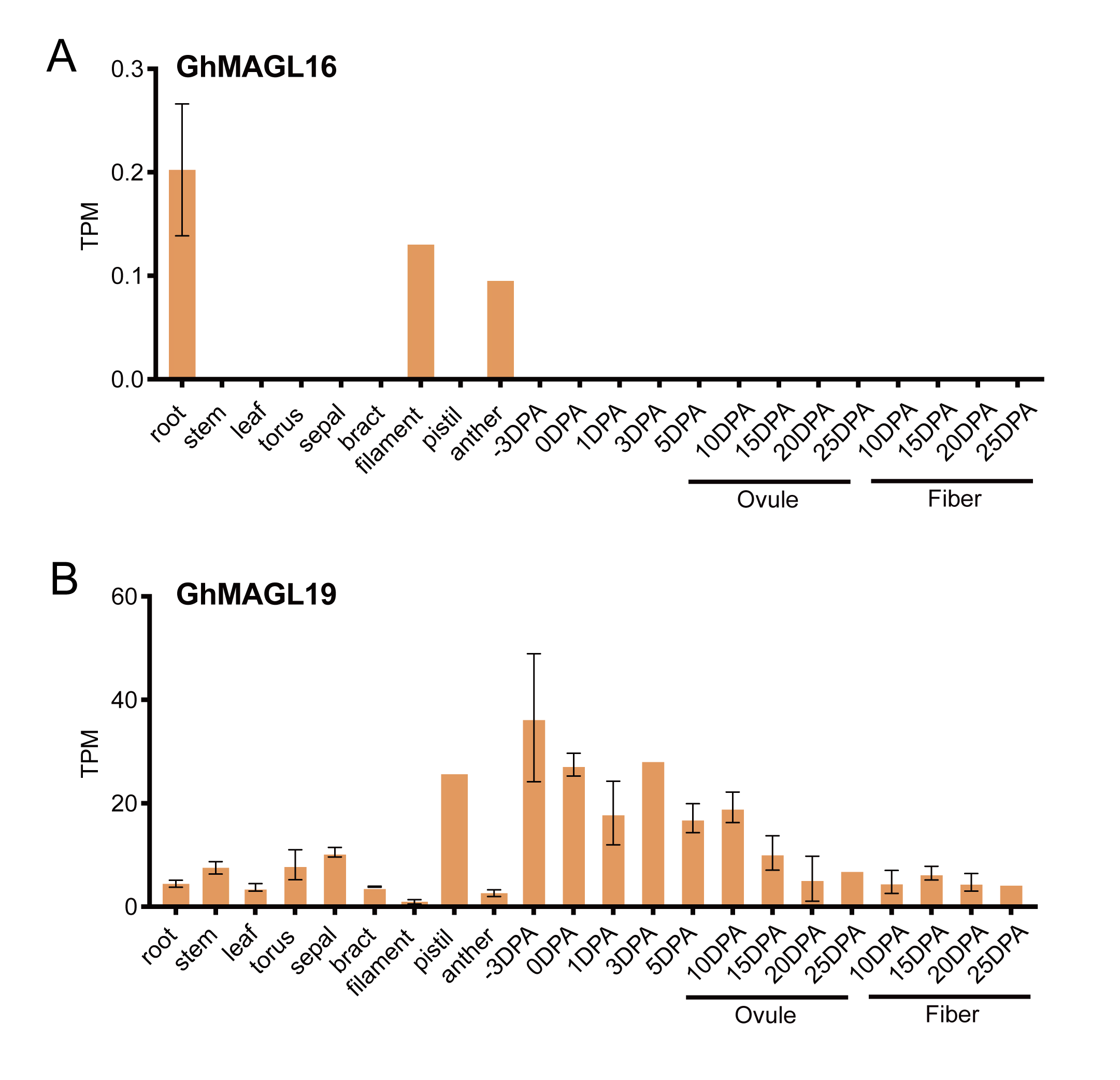


**Figure S5:** The tissue-specific expression patterns of *GhMAGL16* and *GhMAGL19*。
